# What is the difference in proprioception between single condylar arthroplasty and high tibial osteotomy? a comparative study on both knees of the same patient

**DOI:** 10.1186/s13018-023-03965-5

**Published:** 2023-07-06

**Authors:** Hao Ge, Yiwei Huang, Hongsong Yan, Yirong Zeng, Jianchun Zeng

**Affiliations:** 1 Guangzhou University of Chinese Medicine, The First Clinical Medical School, Guangzhou University of Chinese Medicine, Jichang Road 12#, District Baiyun, Guangzhou, Guangdong China; 2 Department of Orthopaedics, The First Affiliated Hospital of Guangzhou University of Chinese Medicine, Jichang Road 16#, District 22 Baiyun, Guangzhou, 510405 Guangdong China; 3 Baiyun Hospital, the First Affiliated Hospital of Guangzhou University of Chinese Medicine, Longqi Road 2#, Renhe Town, Baiyun, Guangzhou, 510405 Guangdong China

**Keywords:** Unicompartmental knee arthroplasty, High tibial osteotomy, FJS-12, Follow-up, Awareness of joint, Arthrophlogosis

## Abstract

**Objective:**

This study aims to investigate the efficacy and outcomes of different surgical procedures, namely unicompartmental knee arthroplasty (UKA) and high tibial osteotomy (HTO), for the treatment of bilateral medial compartment knee osteoarthritis in the same patient. The joint awareness and function of these two surgical methods were evaluated.

**Methods:**

A total of 15 patients with bilateral medial compartment knee osteoarthritis who underwent either UKA or HTO between 2012 and 2020 were included in the study. Patient data, including age, gender, body mass index and length of hospital stay, were collected. Pre- and post-operative measurements were conducted, including tibiofemoral angle, tibial plateau posterior inclination angle, proximal tibial medial angle, distance from mechanical axis to knee joint center, hip-knee-ankle angle, pre- and post-operative knee joint scores, knee joint range of motion, and FIS-12 scores at 3, 6, 12, and 24 months postoperatively. The latest follow-up was used for evaluating the outcomes of osteoarthritis treatment. Normality of continuous variables was assessed using the Shapiro–Wilk test. Between-group comparisons were performed using the paired sample t-test or Wilcoxon rank-sum test. Repeated measures analysis of variance was utilized to analyze FJS-12 measurements at different time points, and the correlation between FJS-12 and postoperative clinical results was examined using Pearson's correlation coefficient. Statistical significance was set at *P* < 0.05.

**Results:**

Significant differences were observed in FJS between the UKA and HTO groups at 3 and 6 months postoperatively, but no significant difference was found at 1 and 2 years postoperatively. FJS in the UKA group demonstrated a significant increase from 3 to 6 months postoperatively, but no significant difference was observed from 6 to 24 months postoperatively. In contrast, FJS in the HTO group showed a significant increase from 3 to 24 months postoperatively.

**Conclusions:**

Patients who underwent UKA exhibited superior joint awareness compared to those who underwent HTO during the early postoperative period. Furthermore, the rate of joint awareness in UKA patients was faster than in HTO patients.

## Introduction

Unicompartmental knee arthroplasty (UKA) and high tibial osteotomy (HTO) have shown improved postoperative functional outcomes compared to total knee arthroplasty when knee degenerative joint disease is limited to the medial compartment [1–3]. HTO is commonly employed as a treatment for medial compartment arthritis of the knee, particularly in young patients. This procedure, first performed in 1958 [4], corrects knee flexion deformity by realigning the mechanical axis of the lower limb [5]. UKA, introduced in the 1970s [6], involves partial joint replacement, preserving the unaffected compartment while addressing the affected compartment. Compared to total knee arthroplasty, UKA offers patients a less invasive surgical option with faster recovery [7].

However, the superiority of one procedure over the other is still under debate. Previous studies have compared the outcomes of the two procedures, with UKA showing better results in terms of function, pain assessment, and complications when compared with HTO [6]. It has been reported that patients treated with UKA achieve higher levels of activity in the early postoperative period compared to those treated with HTO [8]. Nonetheless, studies have also indicated no significant difference in revision rates between HTO and UKA in young patients. Takeuchi et al. highlighted the favorable adaptation of HTO in patients with good knee range of motion [9]. Most previous papers have focused on clinical results, physical activity, pain, and other aspects of the two surgical methods, without evaluating the subjective feeling of patients. Patient-reported outcome measures (PROMs) are commonly employed to assess preoperative and postoperative symptom status in surgical patients. These scores provide a more accurate evaluation of a patient's joint condition and have gained increased attention in recent years due to their ability to reflect patients' own perceptions and satisfaction. The Forgotten Joint Score-12 (FJS-12) has emerged as one of the most widely used PROMs, and it has been validated in multiple languages [10–12].

However, there have been no studies investigating joint awareness after UKA and HTO performed on both knees of the same patient individually. Therefore, the aim of this study is to compare joint awareness and function following different surgical procedures on both knees of the same patient and evaluate the outcomes of these two surgical approaches for the treatment of medial compartment knee arthritis.

## Methods

### Study design and patient selection

This retrospective study received approval from our institutional ethics committee and included 20 consecutive patients who underwent staged UKA or HTO for bilateral knee medial compartment arthritis between 2012 and 2020. The choice of procedure was based on preoperative evaluation.

### Preoperative evaluation

Each involving the medial compartment osteoarthritis of the knee joint in the preoperative assessment. A comprehensive physical examination of the knee joint was conducted for all patients, assessing for tenderness and ligament integrity. In addition, preoperative imaging studies, including X-ray and magnetic resonance imaging (MRI), were performed to evaluate the status of the lateral compartment, patellar joint, and cruciate ligaments. Furthermore, the range of motion (ROM) of the knee joint was measured to assess functional capabilities.

HTO was performed on knees meeting the following criteria: (1) significant knee pain and tenderness; (2) mild medial compartment arthritis (Kellgren-Lawrence grade II and III); (3) varus deformity greater than 5°; (4) absence of significant degeneration in the lateral compartment of the knee joint and the trochlear-femoral joint; (5) no cruciate ligament injury; and (6) absence of severe limitations (flexion contracture less than 5° and knee range of motion ≥ 90°) [13–15].

UKA was performed on knees meeting the following criteria: (1) knee pain and tenderness; (2) moderate to severe involvement of the medial compartment joints (Kellgren-Lawrence grade III and IV); (3) varus deformity less than 15°; (4) absence of significant degeneration in the lateral compartment of the knee joint and the trochlear-femoral joint; (5) no cruciate ligament injury; and (6) absence of severe limitations (flexion contracture less than 5° and knee range of motion  ≥ 90°) [13, 16].

For patients meeting the criteria for both HTO and UKA (Kellgren-Lawrence grade III, varus malformation between 5 and 15°), the doctor and patient engaged in further communication to discuss the characteristics of the two procedures. The final decision regarding HTO or UKA was made by the patient.

### Inclusion and exclusion criteria

Inclusion criteria: Patients with bilateral knee medial compartmental osteoarthritis who underwent either UKA or HTO based on preoperative evaluation.

Exclusion criteria: (1) patients undergoing revision surgery or any additional lower limb surgery; (2) patients with lower limb trauma resulting in pain or limited movement; (3) patients with physical limitations due to other diseases; (4) patients unable to understand and respond to the PROMs used in this study; (5) patients who could not be contacted or refused to participate in the study.

### Surgical procedure

All operations were performed under spinal anesthesia. Based on the preoperative evaluation, UKA was performed on one knee and HTO on the other knee. Bilateral knee surgery was conducted by senior surgeons.

In the UKA group, Oxford unicompartmental knee arthroplasty was performed. The implant placement considered soft tissue balance around the knee joint, maintaining ligaments at resting tension throughout the passive motion range. The minimum residual varus alignment was preserved in the coronal plane. As per the design of the Oxford unicompartmental knee arthroplasty, the planned posterior inclination angle of the tibial component was set at 7° in the sagittal plane for all patients [17].

**Fig. 1:**
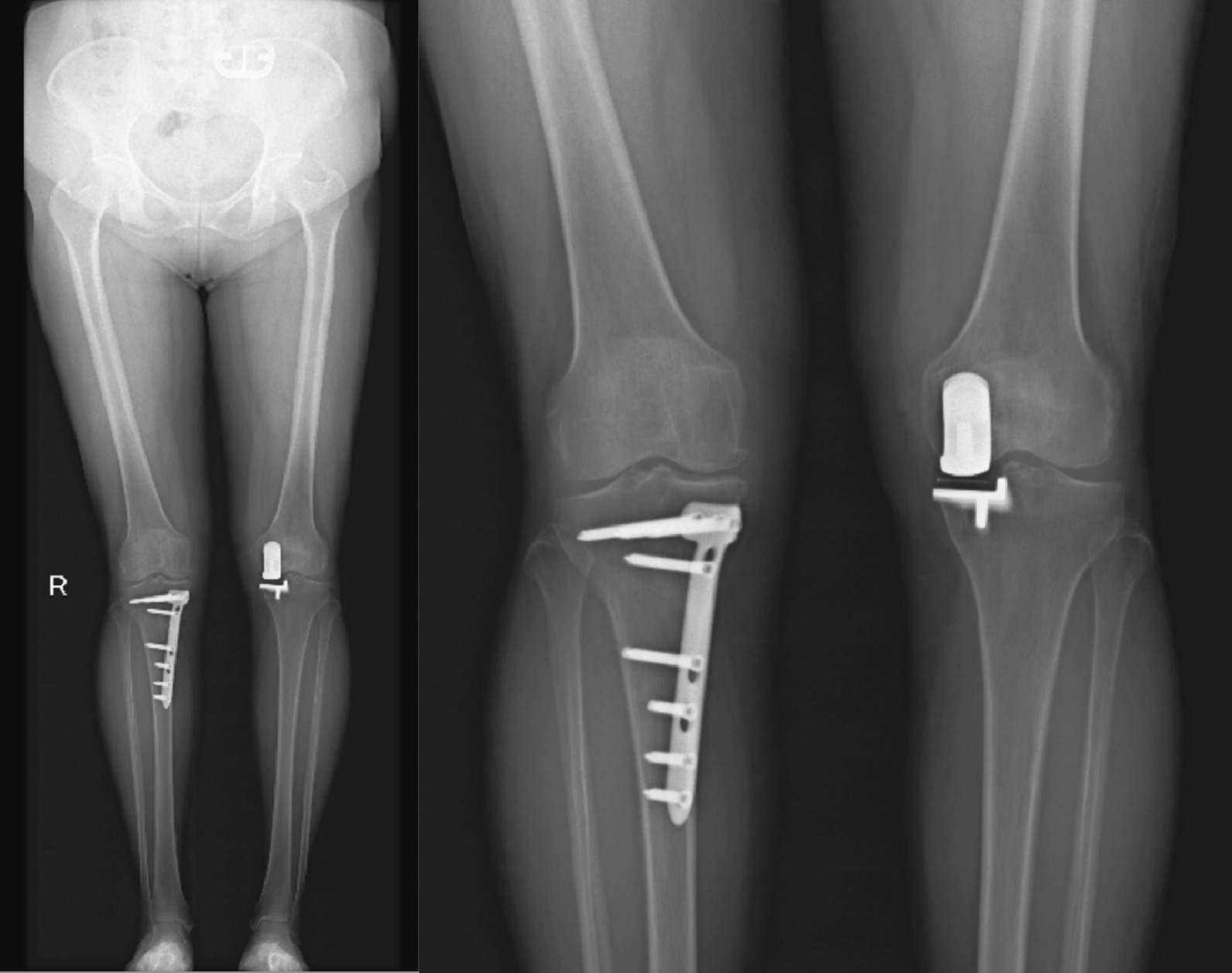
Postoperative images of HTO and UKA. A shows full-length radiographs of the lower limb, and B shows weight-bearing anteroposterior radiographs of the knee joint

In the HTO group, all patients underwent double-plane medial opening wedge high tibial osteotomy to correct varus deformity. Slight overcorrection of varus deformity was necessary to reduce medial compartment pressure, following the description by Fujisawa [18]. The Mikulicz line was planned to pass through the “Fujisawa point” at 62.5% of the entire tibial plateau width measured from the medial side (Fig. 1).

### Rehabilitation after surgery

Patients who underwent HTO were mobilized with caution and permitted to engage in partial weight bearing starting on the second day following the operation. A dedicated rehabilitator supervised the implementation of passive and active ROM exercises, in addition to muscle strengthening and gait rehabilitation training. The transition to walking with a single crutch was initiated at the 1 month mark, while full weight bearing was achieved at six weeks. In the case of UKA surgery, immediate full weight bearing and active assisted ROM exercises of the knee joint were initiated on the second day after the surgical procedure.

### Image data

The following measurements were obtained from full-length, weight-bearing anteroposterior and lateral X-ray films taken before the operation, after the operation, and at the last follow-up: femoraltibial angle (FTA), posterior slope of the tibial plateau (PTS), medial proximal tibial angle (MPTA), distance from the mechanical axis to the center of the knee joint (MAD), and hip-knee-ankle angle (HKA).

### Follow-up

Before the operation, Knee Society score (KSS) and knee range of motion (ROM) were measured. The Forgotten Joint Score-12 (FJS-12) was evaluated using a questionnaire at 3 months, 6 months, 12 months, and 24 months after surgery. At the last follow-up, the Knee Osteoarthritis Outcome Score (KOOS), FJS-12 score, KSS score, and the range of motion of the knee joint were measured. The results were recorded and summarized using Microsoft Excel 2022.

### Statistical methods

Measurements were described using means ± SD or medians. Paired t-tests (Wilcoxon signed-rank test) were used to compare BMI, ROM, KSS, KOOS, FTA, MPTA, MAD, and HKA between the UKA and HTO groups. Repeated measures ANOVA were used to compare preoperative and postoperative joint awareness. Each item of FJS-12 between UKA and HTO was compared, and the results of FJS-12 after UKA and HTO were validated. The relationship between FJS-12 and postoperative clinical outcome was analyzed for each knee using Pearson's correlation coefficient. A p-value less than 0.05 was considered statistically significant. All statistical analyses were performed using SPSS 26.

## Results

Eligibility was assessed in 40 knees from 20 patients treated bilaterally with either UKA or HTO. After excluding patients with adverse events and those who did not respond to the questionnaire, 10 knees from 5 patients were excluded. Three of these patients were not followed up for at least one year, and the other two patients did not have adequate records. Finally, 30 knees from 15 patients, 2 males and 13 females, were analyzed.

### Demographics

The demographic characteristics of the study participants are presented in Table 1. The mean age at the time of initial surgery was 59.6 ± 2.9 years for the UKA group and 60.3 ± 4.2 years for the HTO group (*P* = 0.25). The duration of hospital stay did not differ significantly between the two groups, with a mean of 7.47 ± 1.19 days for the UKA group and 7.5 ± 1.2 days for the HTO group.

**Table 1:** Demographics of patients

Characteristics	UKA	HTO	*P*-value
Number of knees	15	15	
Sex (male/female)	2/13	2/13	
Age at first (years)	59.6 ± 2.9	60.3 ± 4.2	0.250
Length of stay	7.47 ± 1.19	7.5 ± 1.2	
KL classification (%)			
II	0 (0)	11 (73)	
III	9 (60)	4 (27)	
IV	6 (40)	0 (0)	
BMI (kg/m^2^)	26.95 ± 3.58	27.42 ± 10.63	0.270
Preoperative ROM (°)			
Flexion	112.86 ± 17.71	111 ± 14.54	0.673
Extension	1.2 ± 1.5	2.63 ± 1.20	0.745
Preoperative			
KSS-K	54.6 ± 2.85	53 ± 3.273	0.093
KSS-F	64.533 ± 3.87	64.667 ± 4.670	0.941
Postoperative			
KSS-K	84.933 ± 2.987	84.8 ± 2.512	0.907
KSS-F	87.867 ± 1.178	86 ± 2.952	0.185
Postoperative			
KOOS			
Pain	85.941 ± 4.35	87.8067 ± 4.34	0.221
Symptoms	83.668 ± 6.856	85.122 ± 4.731	0.457
ADL	84.371 ± 8.17	85.856 ± 5.4886	0.46
Sports and rec	59.333 ± 7.286	62.666 ± 12.937	0.344
QOL	60.75 ± 10.946	61.2 ± 9.410	0.907

Regarding the preoperative measurements, the mean flexion angle was 112.86 ± 17.71° for the UKA group and 111 ± 14.54° for the HTO group (*P* = 0.67). The mean preoperative extension angle was -1.2 ± 1.5° for the UKA group and 2.63 ± 0.75° for the HTO group. There were no statistically significant differences observed in the postoperative Knee Society Score (KSS) and Knee Injury and Osteoarthritis Outcome Score (KOOS) items between the two groups, as indicated in Table 1.

### FJS

A repeated measure ANOVA was conducted to examine the FJS-12 scores of patients at 3 months, 6 months, 12 months, and 24 months. The results revealed significant changes in FJS scores over time (F_(3, 26)_ = 155.671, *P* < 0.001). The mode of surgery also had a significant impact on FJS scores (F_(1, 28)_ = 14.449, *P* = 0.001 < 0.05). Additionally, there was a significant interaction effect between time and surgical modality (F_(3, 26)_ = 18.038, *P* < 0.001), indicating that the trend of FJS-12 scores varied depending on the treatment modality.

Table 2 illustrates a simple effect analysis of the time trend of FJS-12 in both groups. The UKA group exhibited a significant increase in FJS scores from 3 to 6 months postoperatively, but no significant change in FJS-12 scores from 6 to 12 months postoperatively. Conversely, the HTO group demonstrated a significant increase in FJS scores from 3 months postoperatively to the final follow-up.

**Table 2:** Simple effect analysis of postoperative FJS time trend

(I) Time	(J)Time	UKA			HTO		
		Average difference (I-J)	Standard error	*P*-value	Average difference (I-J)	Standard error	*P*-value
3 months	6 months	− 17.778^*^	1.377	< 0.001	− 21.111^*^	1.377	< 0.001
	12 months	− 18.056^*^	1.477	< 0.001	− 28.056^*^	1.477	< 0.001
	24 months	− 18.333^*^	1.542	< 0.001	− 30.278^*^	1.542	< 0.001
6 months	3 months	17.778^*^	1.377	< 0.001	21.111^*^	1.377	< 0.001
	12 months	0.278	0.741	0.999	− 6.944^*^	0.741	< 0.001
	24 months	0.556	0.842	0.987	− 9.167^*^	0.842	< 0.001
12 months	3 months	18.056^*^	1.477	< 0.001	28.056^*^	1.477	< 0.001
	6 months	0.278	0.741	0.999	6.944^*^	0.741	< 0.001
	24 months	0.278	0.362	0.972	− 2.222^*^	0.362	< 0.001
24 months	3 months	18.333^*^	1.542	< 0.001	30.278^*^	1.542	< 0.001
	6 months	0.556	0.842	0.987	9.167^*^	0.842	< 0.001
	12 months	0.278	0.362	0.972	2.222^*^	0.362	< 0.001

*The significance level of the difference between the means was less than 0.05

Table 3 presents a simple effect analysis of the treatment modality trend of FJS-12 in the two patient groups. FJS scores were significantly higher in the UKA group than in the HTO group at 3 and 6 months postoperatively, but there was no significant difference in FJS scores between the two groups at 1 and 2 years postoperatively.

**Table 3:** Simple effect analysis of trends in postoperative FJS-12 treatment modality

Time	UKA		HTO		*P*-value	Average difference	Standard error
	Mean	SD	Mean	SD			
3 months	51.3889	8	38.0556	6	< 0.001	13.333^*^	2.475
6 months	69.1667	6	59.1667	5	< 0.001	10.000^*^	2.002
12 months	69.4444	6	66.1111	5	0.097	3.333	1.941
24 months	69.7222	6	68.3333	5	0.473	1.389	1.908

*The significance level of the difference between the means was less than 0.05

This study analyzed the differences between individual items of the FJS-12. The results indicated significant differences in the first item (lying in bed at night), third item (walking for more than 15 min), eighth item (standing up from a low seat), ninth item (standing for a long time), and twelfth item (doing preferred exercise) at 3 months, but no significant differences were observed in the remaining seven items. There were no significant differences between the two measures at the last follow-up (Table 4).

**Table 4:** Univariate analysis of FJS in the UKA group versus the HTO group

	3 months			24 months		
	UKA	HTO	*P*	UKA	HTO	*P*
FJS 1	1.9 ± 0.4	3.2 ± 0.4	< 0.001	1.2 ± 0.3	1.3 ± 0.6	0.705
FJS 2	1.8 ± 0.7	2.1 ± 0.7	0.271	1.2 ± 0.4	1.1 ± 0.5	0.705
FJS 3	1.9 ± 0.8	3	0.002	1.3 ± 0.9	1.2 ± 0.6	0.763
FJS 4	1.8 ± 0.6	2.1 ± 0.5	0.206	1.2 ± 0.4	1.2 ± 0.4	1
FJS 5	0.6 ± 0.5	0.8 ± 0.4	0.18	0.4 ± 0.5	0.5 ± 0.5	0.48
FJS 6	2.8 ± 0.4	3	0.083	1.9 ± 0.5	1.6 ± 0.5	0.96
FJS 7	1.9 ± 1.1	2	0.888	1.2 ± 0.7	1.2 ± 0.6	1
FJS 8	1 ± 0.7	1.9 ± 0.9	0.008	0.7 ± 0.6	1 ± 0.4	0.102
FJS 9	0.9 ± 0.5	1.9 ± 0.99	0.005	0.5 ± 0.5	0.9 ± 0.5	0.034
FJS 10	2.8 ± 0.6	3.1 ± 0.6	0.206	1.4 ± 0.5	1.5 ± 0.5	0.655
FJS 11	3.1 ± 0.4	2.9 ± 0.5	0.48	1.5 ± 0.7	1.5 ± 0.5	1
FJS 12	3.2 ± 0.4	3.9 ± 0.4	0.002	2.0 ± 0.02	2.2 ± 0.4	0.083

The validation analysis of FJS assessment results between UKA and HTO at the last follow-up is presented in Table 5. Significant correlations were found between FJS scores and each item of KOOS and KSS in both groups. Regarding range of motion (ROM), knee extension angle showed a significant correlation with FJS scores in the UKA group but not in the HTO group. There was no correlation between knee flexion angle and FJS scores in either the UKA or HTO group. Furthermore, the internal consistency of UKA, as measured by Cronbach's alpha, ranged from 0.85 to 0.94, while for HTO, it ranged from 0.79 to 0.95.

**Table 5:** Association of FJS-12 with other clinical outcomes

	UKA		HTO	
	Correlation	*P*-value	Correlation	*P*-value
KOOS				
Pain	0.678	0.006	0.8	< 0.001
Symptoms	0.793	< 0.001	0.833	< 0.001
ADL	0.623	0.013	0.66	0.007
Sport and rec	0.839	< 0.001	0.603	0.017
QOL	0.745	0.001	0.734	0.002
KSS				
Knee	0.858	< 0.001	0.778	0.001
Function	0.67	0.006	0.554	0.0.32
ROM伸	0.558	0.031	0.13	0.644
ROM曲	0.239	0.39	0.049	0.862
post-FTA	0.239	0.39	0.654	0.008
post-PTS	0.55	0.034	0.034	0.905
post-MPTA	0.54	0.038	0.06	0.831
post-MAD	0.182	0.516	0.709	0.003
post-HKA	0.315	0.253	0.608	0.016

### Imaging

Table 5 presents the correlation between FJS and the magnitude of preoperative and postoperative changes in femoraltibial angle (FTA), posterior slope of the tibial plateau (PTS), medial proximal tibial angle (MPTA), distance from the mechanical axis to the center of the knee joint (MAD), and hip-knee-ankle angle (HKA) in both groups. Notable significant differences were observed in postoperative FTA, postoperative MPTA, preoperative MAD, and HKA between the two groups. In the UKA group, there was no significant association between postoperative FJS and the magnitude of change in preoperative and postoperative FTA, PTA, MPTA, MAD, and HKA. However, in the HTO group, postoperative FJS results showed a positive correlation with the magnitude of change in FTA (*P* = 0.001) and MAD (*P* = 0.047), and a negative correlation with the magnitude of change in HKA (*P* = 0.02).

## Discussion

The FJS-12, initially developed in 2012, is a joint-specific questionnaire that addresses patient awareness and exhibits a lower ceiling effect compared to other PROMs [19, 20]. This characteristic enables it to more effectively differentiate patients achieving good and excellent outcomes. To our knowledge, this is the first study to compare joint awareness in both knees of the same patient undergoing UKA and HTO, respectively.

The HTO group consisted of older patients compared to the UKA group at the time of surgery, although this difference was not statistically significant (Table 1). Given the distinct indications for these procedures, knee osteoarthritis was more severe in the UKA group, predominantly classified as grade III and IV, while the Kellgren-Lawrence (KL) grade in the HTO group ranged from grade II to III.

Previous studies have reported that HTO is superior to UKA in terms of range of motion (ROM) [21]. However, contrary to these findings, our study did not observe a significant difference in preoperative and postoperative ROM between the two groups. Furthermore, the correlation between FJS and postoperative clinical outcomes indicated that FJS in the HTO group was associated with postoperative knee extension, although a causal relationship has yet to be established.

Numerous studies have compared the advantages of UKA and HTO, and our findings align with previous research [22]. Specifically, FJS scores were significantly higher in the UKA group compared to the HTO group at 3 and 6 months, while no significant difference was observed between the two groups at 12 and 24 months (Table 3). These results are consistent with a prior study conducted by Jin et al. [23], which also reported similar FJS outcomes without a significant difference between UKA and HTO at the last follow-up.

**Fig. 2:**
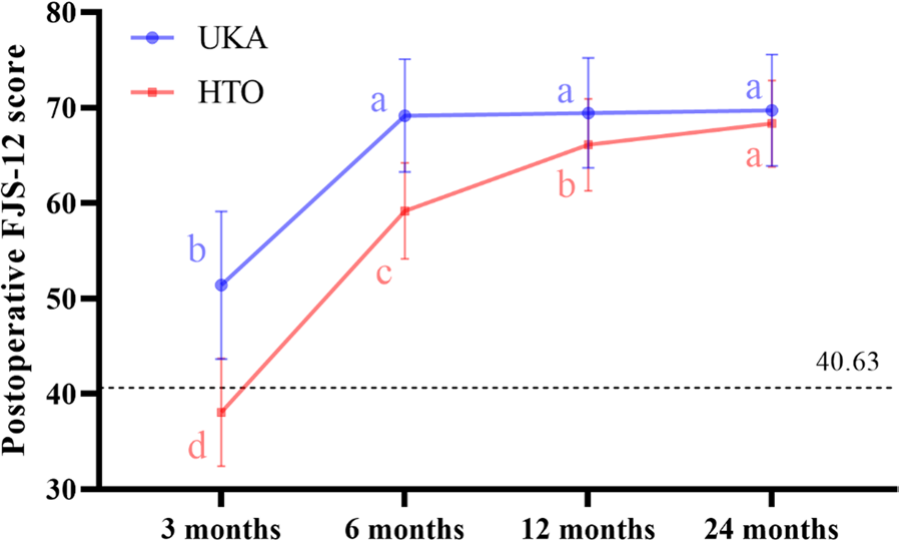
Line plots of the mean FJS over time in both groups. a, b is the time point difference shown by the letter marking method

In the UKA group, there was a significant increase in FJS from 3 to 6 months after surgery, but no significant change was observed from 6 to 24 months post-surgery. Conversely, in the HTO group, FJS-12 showed a significant increase from three months to 24 months after surgery (Fig. 2). These findings indicate that patients in the UKA group experienced a higher degree of joint forgetting in the early stages compared to the HTO group. This may be attributed to the more intense pain experienced during the initial phases of HTO. Studies have shown that patients who undergo UKA generally experience less severe pain than those who undergo HTO, which may contribute to a higher quality of life [24]. Additionally, HTO involves realigning the lower limb, relieving pressure on the medial side of the knee joint. Numerous studies have confirmed that fibrous cartilage regeneration occurs after HTO, and it takes time for the regenerated cartilage to mature [25, 26]. Furthermore, the fixation plate used in HTO may generate friction with the surrounding soft tissues, potentially leading to a decrease in FJS.

There are no studies on the PASS value of FJS after HTO, and a study on Asian UKA showed that the threshold of FJS-12 for PASS was 40.63 (sensitivity: 84.1%, specificity: 76.5%) and 84.38 (sensitivity: 97.1%, specificity: 88.1%) for obliterated joints [27]. The UKA group reached the PASS value at 3 months, while the HTO reached the PASS value at 3 months before the mean FJS reached the PASS value (Fig. 2). Neither group reached the mean FJS at the final follow-up for the forgotten joint. This result may be due to the relatively young patients in this study with higher exercise requirements and greater postoperative expectations.

In the univariate analysis of FJS at 3 months and the final follow-up, patients in the HTO group reported occasional awareness of the artificial joint when lying in bed at night, walking for more than 15 min, and during preferred exercises. Conversely, patients in the UKA group rarely felt the presence of the artificial joint in these situations. Furthermore, the HTO group reported rare awareness of the artificial joint when standing up from a low seat and during prolonged standing, whereas this was barely felt by the UKA group. Consistent with previous studies, the results of FJS in both groups demonstrated high agreement with KOOS scores and KSS scores.

The influence of imaging indexes on postoperative FJS has been a topic of debate. In our study, we found that postoperative FJS in the UKA group was not associated with changes in preoperative and postoperative FTA, PTA, MPTA, MAD, and HKA. In contrast, the HTO group showed a positive correlation with changes in FTA and MAD, while displaying a negative correlation with changes in HKA (Table 5). This discrepancy may be attributed to the fact that HTO alters the force distribution in the lower limb and corrects the inversion deformity.

Previous research has indicated that a high BMI is linked to lower FJS scores following HTO and UKA. To eliminate confounding factors, such as age, gender, and BMI, we focused on the bilateral knee joints of the same patients in our study. By comparing the forgotten joints of patients who underwent UKA and HTO for medial knee osteoarthritis, we aimed to provide a more objective demonstration of the differences. This approach can serve as a valuable reference for clinicians when selecting surgical procedures in clinical practice. Additionally, the observed trends in postoperative FJS can influence the formulation of rehabilitation plans for patients.

In summary, these findings contribute to our understanding of the differences in forgotten joint perception following UKA and HTO procedures. The results suggest that UKA patients experience a faster recovery and a reduced impact on their quality of life. Moreover, the degree of forgotten joints in UKA patients appears to be unrelated to specific imaging indexes, whereas in HTO patients, it is influenced by changes in FTA, MAD, and HKA. These insights can assist clinicians in selecting appropriate surgical interventions and guide the development of tailored rehabilitation plans for patients.

Nevertheless, several limitations should be acknowledged in this study. Firstly, the sample size might be relatively small, which could impact the generalizability of the findings. Secondly, the follow-up period was relatively short, limiting our ability to assess and compare long-term outcomes between the two procedures. Consequently, further studies with larger sample sizes and longer follow-up periods are warranted to evaluate the sustained effects of these surgical interventions.

## Conclusion

The present study revealed a robust correlation and internal consistency between the FJS and the Knee injury and Osteoarthritis Outcome Score (KOOS) and Knee Society Score (KSS) in both UKA and HTO patients. Furthermore, the degree of forgotten joints was found to be higher in early-stage UKA patients compared to those undergoing HTO, but this discrepancy converged at the one-year postoperative mark. Additionally, UKA patients experienced a shorter postoperative recovery period, resulting in less impact on their daily lives. Notably, the degree of forgotten joints in UKA patients was independent of the extent of change in FTA, PTA, MPTA, MAD, and HKA. In contrast, among HTO patients, the degree of forgotten joints exhibited a positive correlation with changes in FTA and MAD, while displaying a negative correlation with changes in HKA.

## Data Availability

The authors declare that all the data supporting the findings of this study are available within the article.
